# Intra and inter-rater reliability study of pelvic floor muscle dynamometric measurements

**DOI:** 10.1590/bjpt-rbf.2014.0083

**Published:** 2015-04-27

**Authors:** Natalia M. Martinho, Joseane Marques, Valéria R. Silva, Silvia L. A. Silva, Leonardo C. Carvalho, Simone Botelho

**Affiliations:** 1 Laboratório de UroFisioterapia, Curso de Fisioterapia, Escola de Enfermagem, Universidade Federal de Alfenas (UNIFAL-MG), Alfenas, MG, Brazil; 2 Departamento de Cirurgia, Faculdade de Ciências Médicas, Universidade Estadual de Campinas (UNICAMP); Campinas, SP, Brazil

**Keywords:** muscle strength dynamometer, pelvic floor, physical therapy, reproducibility of results

## Abstract

**OBJECTIVE:**

The aim of this study was to evaluate the intra and inter-rater reliability of pelvic floor muscle (PFM) dynamometric measurements for maximum and average strengths, as well as endurance.

**METHOD:**

A convenience sample of 18 nulliparous women, without any urogynecological complaints, aged between 19 and 31 (mean age of 25.4±3.9) participated in this study. They were evaluated using a pelvic floor dynamometer based on load cell technology. The dynamometric evaluations were repeated in three successive sessions: two on the same day with a rest period of 30 minutes between them, and the third on the following day. All participants were evaluated twice in each session; first by examiner 1 followed by examiner 2. The vaginal dynamometry data were analyzed using three parameters: maximum strength, average strength, and endurance. The Intraclass Correlation Coefficient (ICC) was applied to estimate the PFM dynamometric measurement reliability, considering a good level as being above 0.75.

**RESULTS:**

The intra and inter-raters' analyses showed good reliability for maximum strength (ICC_intra-rater1_=0.96, ICC_intra-rater2_=0.95, and ICC_inter-rater_=0.96), average strength (ICC_intra-rater1_=0.96, ICC_intra-rater2_=0.94, and ICC_inter-rater_=0.97), and endurance (ICC_intra-rater1_=0.88, ICC_intra-rater2_=0.86, and ICC_inter-rater_=0.92) dynamometric measurements.

**CONCLUSIONS:**

The PFM dynamometric measurements showed good intra- and inter-rater reliability for maximum strength, average strength and endurance, which demonstrates that this is a reliable device that can be used in clinical practice.

## Introduction

Pelvic floor muscle (PFM) evaluation is recommended by the International Continence Society (ICS) and considered essential to evaluate a post-therapeutic intervention effect^1^. Several methods are used by different researchers, among them vaginal dynamometry has been particularly investigated throughout scientific fields^2^^-^ 11. According to Dumoulin et al.^12^, vaginal dynamometry can be an efficient tool for the direct investigation of female PFM strength.

Following the earlier models of vaginal dynamometers^12^^-^ 14, other devices have been developed. Dumoulin et al.^12^ developed the Montreal dynamometer, capable of measuring PFM strength in Newtons (N), and used it in several studies^2^^-^ 5 ^,^ 11. This instrument has been improved over the years, allowing it to assess dynamometric measurements of the PFM's passive properties^6^^,^ 8, speed of contraction, and endurance^5^. Saleme et al.^7^ developed a dynamometric speculum which can measure PFM strength multidirectionally, according to vaginal canal morphology. These intravaginal devices, however, vary as to size, shape, force vector (anteroposterior, lateral or multilateral force), and other technical characteristics^7^^,^ 9 ^,^ 12 ^,^ 15 ^-^ 18.

Studies using vaginal dynamometers showed a good ability and repeatability of measuring PFM strength^2^^-^ 4 ^,^ 15, with test-retest reliability^2^^,^ 6 ^,^ 9 ^,^ 17 ^,^ 18 as well as ability to investigate other pathophysiological parameters such as endurance, speed of contraction, and muscle tone^5^^,^ 8 ^,^ 10 ^,^ 18.

However, the main limitation associated with PFM dynamometers is their lack of accessibility because these devices are mostly used by their designers and are not commercially available, a fact which excludes measurement reproducibility. Thus, this study proposed to investigate the intra and inter-rater reliability of PFM dynamometric measurements for maximum and average strengths, as well as endurance, using an equipment locally available.

## Method

### Study design

This was a test-retest study, assessing intra- and inter-rater reliability of PFM dynamometric measurements.

### Participants

A convenience sample of 18 nulliparous women, without any urogynecological complaints, aged between 19 and 31 (mean age of 25.4±3.9) participated in this study. All participants signed an informed consent form, and the study was approved by the research ethics committee of Universidade Federal de Alfenas (UNIFAL-MG), Alfenas, MG, Brazil (CAAE: 06620512.4.0000.5142). The inclusion criteria were: nulliparous women, between 18 and 35 years old, normal body mass index (<25 kg/m^2^), without any urogynecological complaints and presenting PFM strength equal to or greater than grade 1, according to the *Modified Oxford Grading Scale* 19. The exclusion criteria were: pregnant women, pelvic organ prolapse or reconstructive pelvic surgery, symptoms of vaginal infection, intolerance to condoms, allergy to the gel used in the procedure, degenerative neurological disorder or any other disease that may interfere with PFM strength measurements, being in either a pre-menstrual or current menstrual period^2^^,^ 5 ^,^ 20.

### Assessment tools

A dynamometer designed to measure PFM strength was used in the present study (*EMG System do Brasil, model DFV 020101/10*(r)). The vaginal dynamometer is cylindrical in shape (9.5cm in length and 3.3cm in diameter), made externally in plastic and internally in steel structures and equipped with a load cell 2cm from its base, which can measure anteroposterior unidirectional compressive strength in kilogram/force (Kgf) units. The vaginal dynamometer was connected to a computer and both remained unplugged from the mains during the collections to avoid any interference.

### Interventions

**Figure 1.**
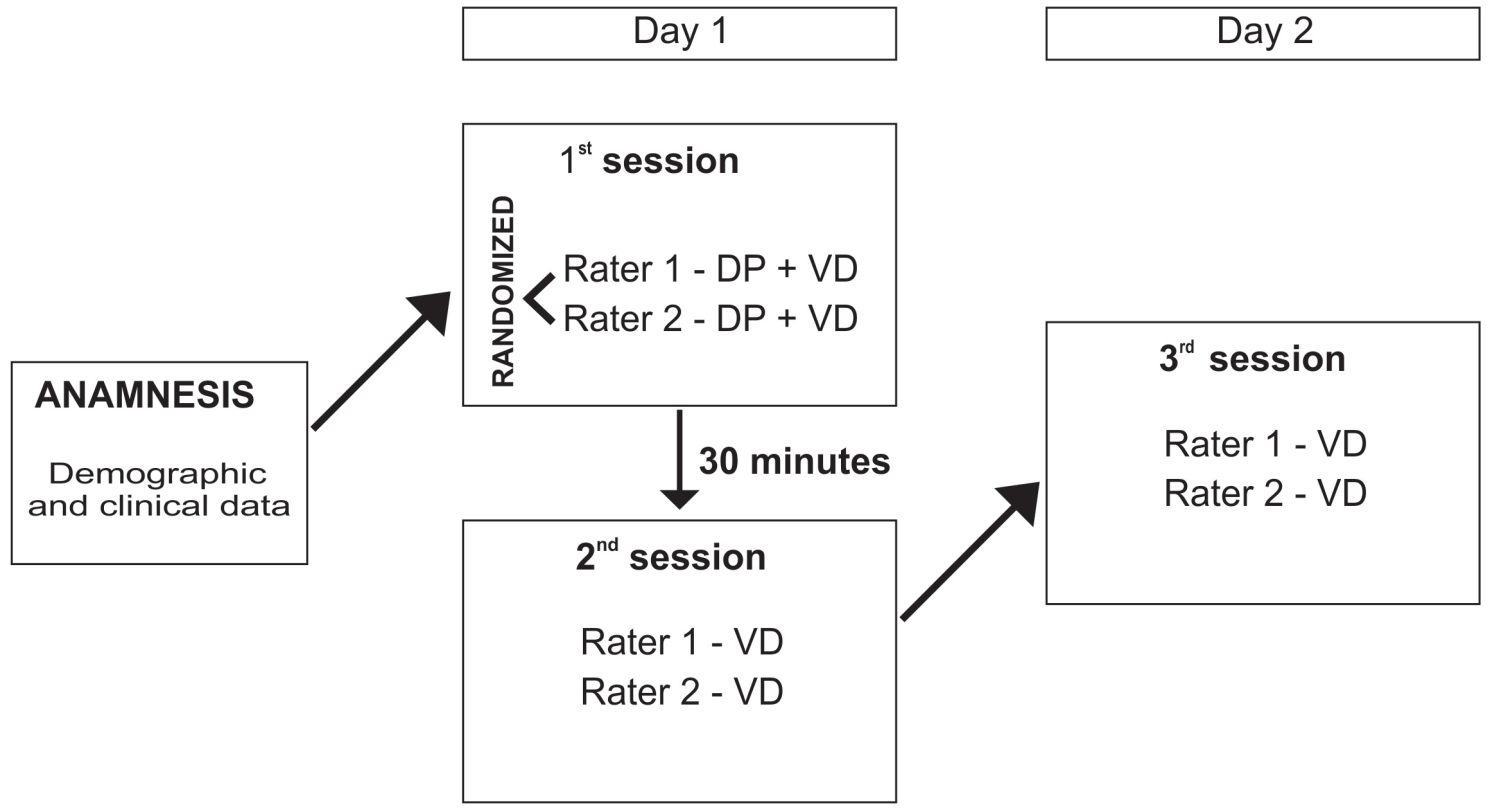
Methodology chart used for PFM assessment. The above chart presents the methodology used for PFM assessment. In the first session, the order between raters was randomly selected and then maintained during the following sessions. PFM: Pelvic floor muscles; DP: digital palpation; VD: vaginal dynamometry.

PFM strength was evaluated for all women and repeated in three successive sessions: two on the same day with a rest period of 30 minutes between them, and the third on the following day. First, an interviewer asked the participants to provide their demographic and clinical data. Then, all participants were evaluated twice in each session, first by examiner 1 followed by examiner 2, in a randomly selected order, as presented in Figure 1. The interviewer remained in the assessment room to ensure that the same procedures were performed by both raters and the raters were blinded to each other's results.

As in Ferreira et al.^20^, both examiners in this study were previously trained to perform the PFM assessment protocol (digital palpation and dynamometric assessment) by a well-experienced physical therapist with 16 years of clinical practice experience. They also had comprehensive knowledge and experience in PFM assessment skills.

The ability to contract and relax the PFM was first evaluated by digital palpation, in the lithotomy position. The participant was asked to perform a maximum contraction of her PFM, lifting it inward and squeezing around the fingers then completely relaxing it^20^. When a correct contraction was verified, the examiner scored it according to the *Modified Oxford Grading Scale* (0-5 points)^19^, which determined the participant's eligibility.

Thus, PFM strength was assessed with the vaginal dynamometer, which was covered with a condom (*Elite*(r)) and lubricated with hypo-allergenic gel (*Johnson & Johnson*(r)* KY gel*), then inserted into the vaginal cavity with the load cell positioned so that it could capture the anteroposterior compression strength. Next, the participant was asked to perform three maximal voluntary PFM contractions, recorded for 15 seconds with a rest period of three minutes after each one of them^21^ directed by a verbal command as follows: *"When I ask you, please, perform a pelvic floor contraction as hard as possible, maintaining as long as you can and then relax when you get tired".*

### Data analysis

**Figure 2.**
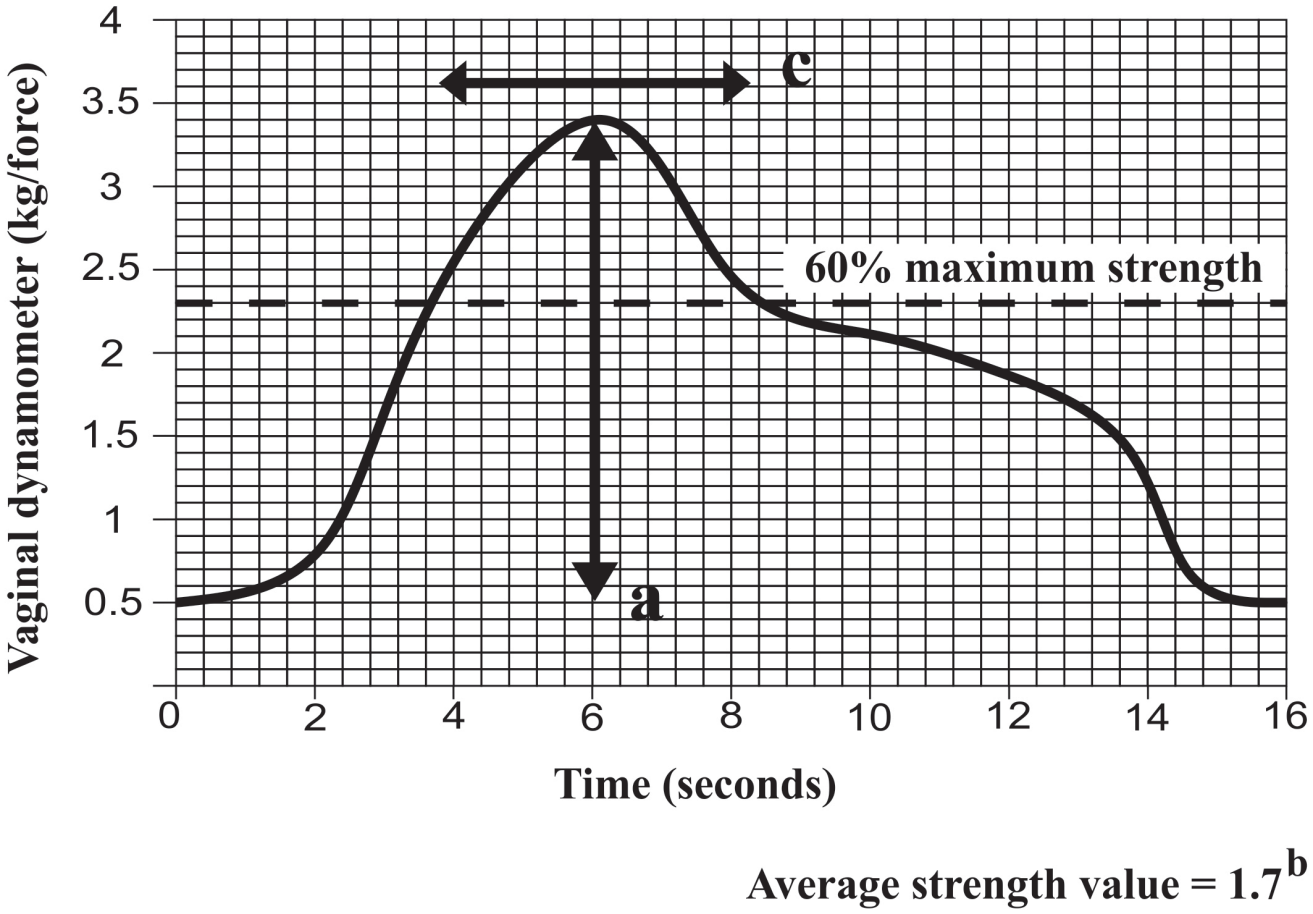
Illustration of the parameters used to analyze vaginal dynamometry data. a: Maximum strength: calculating the difference between the highest and lowest strength values; b: Average strength: a mean value of the strength curve; c: Endurance: equal to the length of time during which the participant could maintain a contraction above 60% of her maximum strength. Kgf: Kilogram/force.

The vaginal dynamometry data were analyzed by the main researcher, using three parameters (Figure 2):

- *Maximum strength*: the researcher calculated the difference between the highest and lowest strength values, which were provided by the equipment software^3^, in kgf*.*
- *Average strength*: a mean value of the strength curve, provided by the equipment software, in kgf*.*
- *Endurance*: equal to the length of time, in seconds (*s*), during which the participant could maintain a contraction above 60% of her maximum strength^22^^,^ 23.

An average value was calculated for each parameter, using the results of the three values.

### Statistical analysis

Demographic and clinical data were presented as frequency and percentage variables. The intra-rater agreement was analyzed using a type 3,3 Intraclass Correlation Coefficient assessing the measure consistency by each rater in three evaluations. Inter-rater agreement was analyzed using a type 3,1 Intraclass Correlation Coefficient, considering the between-rater concordance during the three sessions using only an average value obtained from the three measures assessed in each session. The following values, suggested by Portney and Watkins^24^ were considered: ˃0.75 = good; from 0.5 to 0.75 = moderate and ˂0.5 = poor.

Moreover, the Standard error of measurement (SEM) and Minimal detectable difference (MDD) were calculated for both intra- and inter-rater reliability analysis, and an inter-rater measurement dispersion study was performed using Bland-Altman plots with limits of agreement.

The *Statistical Package for Social Sciences *(SPSS) 17.0 was used.

## Results

Most participants were single (94.4%), white (94.4%), with complete/incomplete tertiary education (100%), and without any paid labor activity (61.1%). The participants reported using oral contraceptives (77.8%), not having any physical activity (61.1%), and maintaining regular sexual activity (72.2%). The participants' average age was 25.4 (±3.9) years and the average body mass index was 22.9 (±2.9) kg/m^2^.

The digital palpation evaluation showed that all participants presented effective and conscious PFM contractions, which were classified as *strength grade 3* (n=9), *strength grade 4* (n=8), and *strength grade 5* (n=1), using the *Modified Oxford Grading Scale.*

Tables 1 and 2 show the intra- and inter-rater analyses for the dynamometric measurements, respectively.

**Table 1.** Intra-rater reliability of the dynamometric measurements.

	1^st^ session M (SD)	2^nd^ session M (SD)	3^rd^ session M (SD)	Intra-rater reliability (ICC)	Level^*^	CI 95%	SEM	MDD
RATER 1								
Maximum strength (kgf)	1.01 (0.5)	1.03 (0.6)	1.07 (0.6)	0.96	Good	0.79-0.96	0.10	0.28
Average strength (kgf)	0.41 (0.2)	0.42 (0.2)	0.45 (0.3)	0.96	Good	0.92-0.99	0.05	0.13
Endurance (seconds)	3.9 (1.7)	3.95 (2.0)	3.99 (2.2)	0.88	Good	0.73-0.95	0.67	1.86
RATER 2								
Maximum strength (kgf)	1.06 (0.6)	1.11 (0.6)	1.11 (0.7)	0.95	Good	0.89-0.98	0.13	0.59
Average strength (kgf)	0.45 (0.2)	0.46 (0.2)	0.49 (0.3)	0.94	Good	0.87-0.98	0.06	0.28
Endurance (seconds)	3.87 (2.1)	4.21 (2.4)	4.58 (2.1)	0.86	Good	0.70-0.94	0.80	2.20

The table presents the result consistency for each rater, during the three assessment sessions. The mean (M) as well as standard deviation (SD) of the values obtained in each assessment session and by each rater are presented, in addition to the Intraclass Correlation. Coefficient (ICC3,3), Confidence Interval (CI), Standard error of measurement (SEM), and Minimal detectable difference (MDD). Kgf = Kilogram force.

* Portney and Watkins24

**Table 2.** Inter-rater reliability of the dynamometric measurements

	Rater 1 M (SD)	Rater 2 M (SD)	ICC*	CI 95%	SEM	MDD
1^st^ session						
Maximum strength (kgf)	1.01 (0.51)	1.06 (0.55)	0.80	0.54-0.92	0.24	0.65
Average strength (kgf)	0.41 (0.22)	0.45 (0.24)	0.83	0.59-0.93	0.10	0.27
Endurance (seconds)	3.90 (1.69)	3.85 (2.02)	0.59	0.20-0.83	1.17	3.23
2^nd^ session						
Maximum strength (kgf)	1.03 (0.56)	1.12 (0.58)	0.91	0.77-0.96	0.17	0.48
Average strength (kgf)	0.42 (0.23)	0.46 (0.23)	0.88	0.71-0.95	0.08	0.22
Endurance (seconds)	3.95 (1.95)	4.21 (2.35)	0.71	0.38-0.88	1.14	3.16
3^rd^ session						
Maximum strength (kgf)	1.07 (0.56)	1.11 (0.66)	0.87	0.69-0.95	0.22	0.60
Average strength (kgf)	0.45 (0.28)	0.50 (0.30)	0.89	0.74-0.96	0.09	0.26
Endurance (seconds)	3.99 (2.22)	4.58 (2.08)	0.81	0.59-0.93	0.92	2.55

The table presents the agreement between rater 1 and rater 2 during the three assessment sessions. The mean (M) as well as standard deviation (SD) of the obtained values in each assessment session and by each rater are presented, in addition to the Intraclass Correlation Coefficient (ICC3,1), Confidence interval (CI), Standard error of measurement (SEM), and Minimal detectable difference (MDD). Kgf = Kilogram force.

* Portney and Watkins24

**Figure 3.**
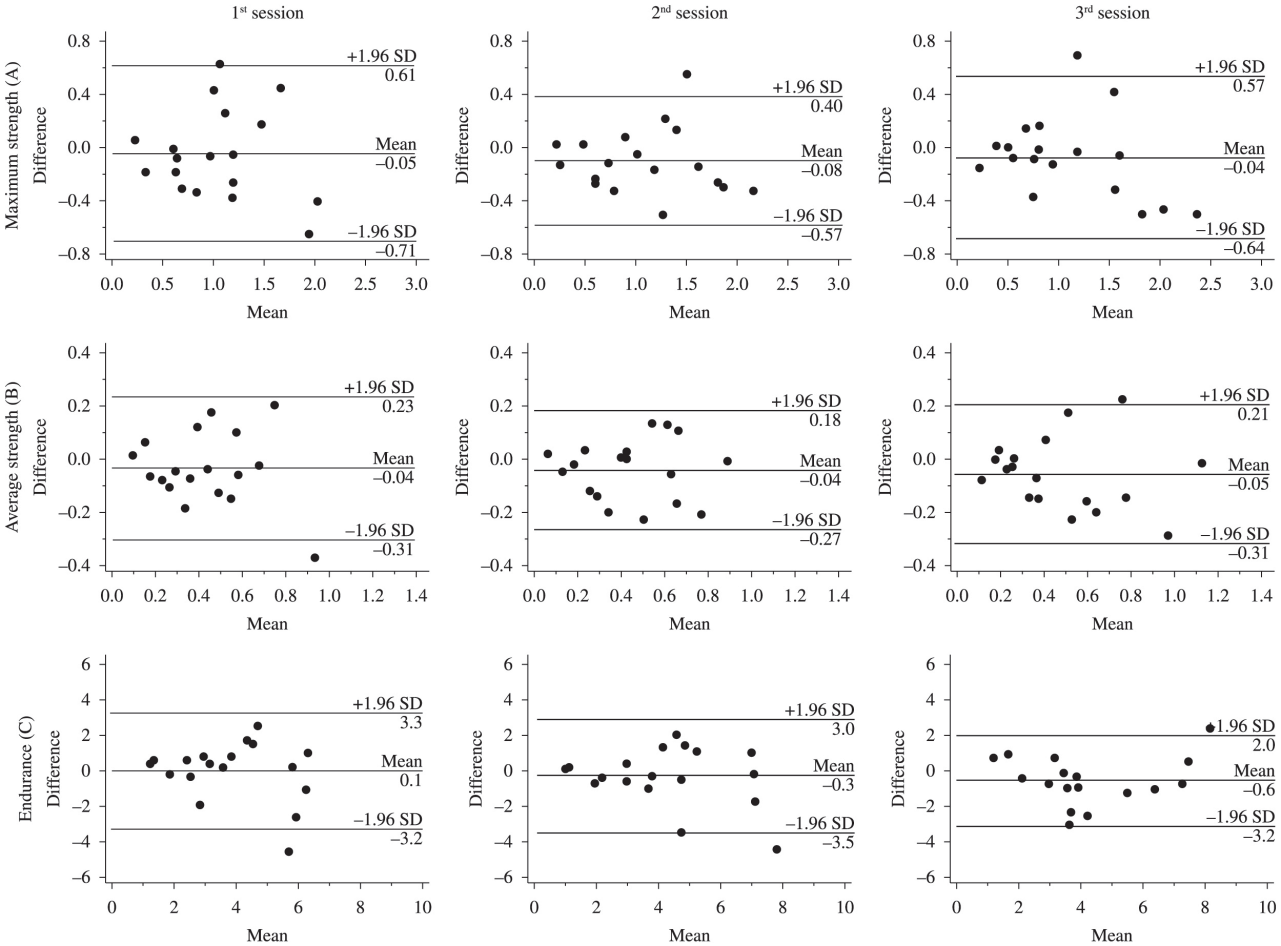
Bland-Altman plots for both raters. The figure shows the dispersion graphs for the analyzed parameters - maximum strength (A), average strength (B), and endurance (C) - considering the inter-rater measures. The means of both raters (X+Y)/2 are presented on the X axis, while the difference between them (bias: X-Y) is presented on the Y axis. It can be noted that the limits of agreement are established (difference ±1.96 SD) and that the majority of the values found (95%) are distributed within this limit.

Figure 3 shows the Bland-Altman plots for both raters.

## Discussion

According to the ICS^25^, PFM function can be qualitatively defined by the tone at rest and the strength of a voluntary or reflex contraction as strong, weak or absent or by a validated grading system. Digital palpation has been used in clinical practice although many researchers do not consider it reliable, objective or sensitive. Several authors who researched its correlation with other methods considered it objective, nevertheless its reproducibility still remains questionable^21^.

Other methods have been used during clinical trials in order to quantify the subjective findings of digital palpation assessment. Among them are: electromyography, perineometry, dynamometry, ultrasound, and magnetic resonance imaging. However, due to the lack of a gold standard for the assessment of women's PFM function, any comparison among the results becomes more difficult and even inaccurate.

Thus, the use of PFM functional assessment is necessary not only to investigate the muscular response, but also to quantify muscle strength^2^, endurance^2^^,^ 5 ^,^ 22 ^,^ 23, speed of contraction^5^, as well as the ability to perform, then repeat, fast and slow contractions^4^.

The protocol used for data analysis in this study was based on previous studies which used different PFM evaluation methods^3^^,^ 22 ^,^ 23 due to the fact that no other study using vaginal dynamometer equipped with a load cell was found in the literature. Thus, three different parameters were analyzed: maximum strength (kgf), average strength (kgf), and endurance (s).

Considering the histological composition of the PFM, composed of approximately 70% type I fibers (slow fibers - responsible for pelvic organ support) and 30% type II fibers (fast fibers - responsible for urethral closure during activities which trigger an increase in intra-abdominal pressure)^26^, both equally important for the maintenance of continence mechanisms^27^, it is believed that the proposed parameters in this study allow a better understanding of muscle function in its totality. So, while clinically evaluating a patient, it is important not only to assess a maximal voluntary contraction but also the ability to maintain a sustained one. Of course, in order to use any device in clinical research, it is essential to verify and analyze its reliability, without which, it would be impossible to rely on the collected data^2^^,^ 28.

The reliability of any PFM evaluation provides basic information about the degree of error within its measurements. The test-retest reliability verifies the stability of repeated measurements performed along different and separate periods of time. Repeated applications may be obtained by multiple evaluations within the same session (intra-session reliability), measurements taken over longer periods of time (test-retest reliability) or comparing the results of different raters (inter-rater reliability)^28^^,^ 29.

There is also a diversity of protocols used among researchers^2^^,^ 5 ^,^ 20 while testing the reliability of PFM measurements. Morin et al.^5^ tested the test-retest reliability of PFM dynamometric measurements using the Montreal dynamometer^12^, by means of two parameters: speed of contraction and endurance. To calculate the speed of contraction, the authors quantified the force rate in the first contraction and the number of fast contractions performed. To analyze the endurance parameter, the authors calculated the area between 10 and 60 seconds under the force curve of a maximal voluntary contraction.

In the present study, as well as in Quartly et al.^22^, the endurance parameter was analyzed considering the time factor (in seconds), measuring the time during which the participant could maintain a contraction above 60% of her maximum strength.

It is common as well as important to verify the time of a sustained contraction in clinical practice. While Quartly et al.^22^ found an average of 5.5 (range 4 to 12) seconds for women under 40 years using a perineometer, the present study found an average of 4.08 (range 1.5 to 9.67) seconds using a vaginal dynamometer.

Two other parameters were also used to quantify PFM strength: maximum strength, also used by Morin et al.^3^ in their study, and average strength, which was proposed as an additional parameter to equalize the findings of fast and sustained PFM contractions.

Another methodological feature to be considered refers to the time interval which comes between an assessment and another one due to the influence of the patient's menstrual cycle, as well as the ability to learn and train performing PFM contractions from one evaluation to the next, which could compromise the comparison^20^. Sigurdardottir et al.^30^ reported that the time range of test-retest reliability performance should be, at most, up to seven days. Thus, in this study, an interval of one day between assessments was determined.

A limitation of the study was that the equipment used in this study has a cylindrical shape 3.3cm wide that can cause some vaginal discomfort and thus interfere with the performance measures, a fact that was also reported by other authors^2^^,^ 7. Another limitation of this equipment would be the difficulty to use it in different positions, as well as with women who suffer from vaginal stiffness.

The use of the vaginal dynamometer has the advantage of quantifying clinical data observed during PFM contraction evaluation and can be used in scientific research, despite its high cost which can be another limiting factor, and in clinical practice. In addition, this model can be protected with a condom followed by disinfection, which facilitates the clinical routine, since it does not need to be privately used or go through a sterilization process, like endovaginal probes which are used in electromyography.

It is known that the larger the sample size is, the greater its consistency and the greater the agreement among the findings will be, ensuring the study's reliability^28^. Accordingly, a higher number of participants would have enforced the present study's findings. Therefore, the PFM dynamometric measurements showed good intra- and inter-rater reliability for maximum strength, average strength, and endurance, demonstrating this to be a reliable device, which can be used in clinical practice.
